# Preoperative detection of insulinomas: two case reports

**DOI:** 10.1186/1757-1626-1-362

**Published:** 2008-11-29

**Authors:** Mesut Ozkaya, Mehmet Fatih Yuzbasioglu, Irfan Koruk, Erman Cakal, Mustafa Sahin, Basak Cakal

**Affiliations:** 1 Department of Endocronology and Metabolism, Sutcu Imam University Medical Faculty, Kahramanmaras, Turkey; 2 Department of General Surgery, Sutcu Ýmam University Medical Faculty, Kahramanmaras, Turkey; 3 Ministry of Health, Department of Gastroenterology, Gaziantep State Hospital, Gaziantep, Turkey; 4 D epartment of Endocronology and Metabolism, Yuksek Ihtisas Hospital, Ankara, Turkey; 5 Ministry of Health, Department of Endocrinologym, Gaziantep State Hospital, Gaziantep, Turkey; 6 Department of Gastroenterology, Yuksek Ihtisas Hospital, Ankara, Turkey

## Abstract

**Background:**

Insulinoma is the most common endocrine tumor of the pancreas. Accurate preoperative detection and localization of insulinomas is essential for the appropriate selection of candidates for surgery. We present two cases with benign pancreatic insulinoma.

**Case presentation:**

Preoperative evaluation for patients with suspected insulinomas has been controversial. Endoscopic ultrasonography (EUS) has a sensitivity of 95% in well skilled operators and well tolerated preoperative imaging method.

**Conclusion:**

We have detected the insulinomas with EUS before surgery in our patients but other imaging modalities did not help us for localization of them. Patients have been asymptomatic postoperatively with no hypoglycemia on repeat fasting. We reviewed here the different modalities for preoperative localization of insulinoma.

## Background

Insulinoma is a neuroendocrine tumour derived mainly from the pancreatic islet cells producing excessive amounts of insulin. Insulinoma is the most common form of the pancreas islet cell tumors (60%). It can be seen at every age but mostly seen in females above age 50 years old [1,2]. Ninety percent of all cases are benign, solitary and located in pancreas. In adults 90% of solitary lesions are smaller than 2 cm, and 30% are smaller than 1 cm in size. Small amount of cases (10%) can be found malignant and multiple. Because of nonspecific symptoms 20% of cases are misdiagnosed primarily. Major symptoms and signs are related to hypoglycemia and become significant due to exercise and fasting. Insulinoma is diagnosed with he elevated (or non-suppressed) insulin levels when hypoglycemia occurred [3].

In cases that were clinically suspected for insulinoma, C-peptide suppression test or prolonged supervised fast tests are used for diagnosis. In these cases, suspected tumor localization has been done with different laboratory and scanning procedures. Because they are small tumors the sensitivity of transabdominal ultrasonography (TAUSG) and abdominal computerized tomography (CT) scanning are low (between 23–63% and 40–73% respectively). Angiography and transhepatic venography have higher sensitivity rates (60–98%) but they are expensive and invasive procedures [2]. Endoscopic ultrasonography (EUS) has a sensitivity of 95% in well skilled operators. Intraoperative ultrasonography (IOUSG) is a well diagnostic era with high sensitivity (90%) but it is difficult to do and it needs pancreatic mobilization and this can cause splenic vein trauma [4]. In this manuscript, we mention two insulinoma cases that were diagnosed in six months period.

## Case 1

**Figure 1:**
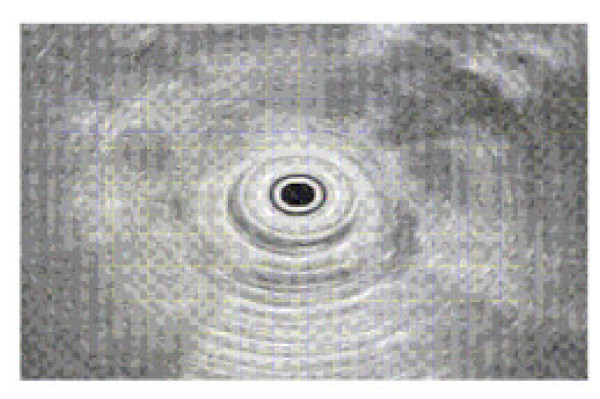
In the EUS scanning at the pancreas uncinate process, hypoechoic 13 mm nodular lesion.

A 43 years old female patient defines hypoglycemic attacks for two years. In physical examination the body mass index (BMI) was 27 kg/m^2^, initial glucose level was 48 mg/dl (70–11 mg/dl), plasma insulin level was 16 μIU/ml (5–25 μIU/ml), C-peptide level was 2.7 ng/ml (1.6–3.6 ng/ml). Prolonged supervised fast test was applied and at the sixteenth hour of the test hypoglycemic symptoms were occurred. Simultaneously taken blood samples showed the glucose 23 mg/dl, plasma insulin 12.7 μIU/ml, insulin/glucose ratio was 0.52. The TAUSG and CT scan showed no pathology. In the EUS scanning at the pancreas uncinate process, there was a hypoechoic 13 mm nodular lesion (Figure 1). It was in homogenous echo and well shaped. It was toughed as neuroendocrine tumor. She was examined for multiple endocrine neoplasia. Magnetic resonance imaging (MRI) of hypophysis, hypophysial hormonal analysis and intact parathormone levels were normal. She was scheduled for surgical operation and preoperatively fluid therapy with dextrose in water and 50 μg (tid) octreotide was applied subcutaneously. In the surgical operation the tumor was found at the localization defined by EUS and enucleation procedure was applied. In the histopathologic examination the specimen showed positive staining for insulin and it was in 15 × 11 × 8 mm in size. The glucose and insulin levels were normal at postoperative period.

## Case 2

**Figure 2:**
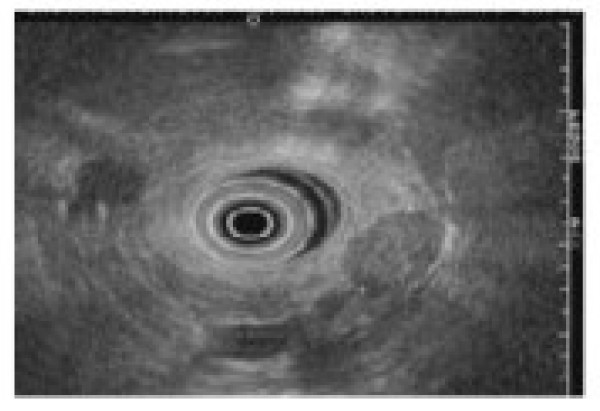
In the EUS scanning at the pancreas uncinate process, hypoechoic 11 mm nodular lesion.

A 62 years old female patient defines hypoglycemic attacks for 1.5 years. In physical examination the body mass index (BMI) was 24 kg/m^2^, initial glucose level was 41 mg/dl (70-1-mg/dl), plasma insulin level was 17 μIU/ml (5–25 μIU/ml), C-peptide level was 12.9 ng/ml (1.6–3.6 ng/ml). Prolonged supervised fast test was applied and at the sixth hour of the test hypoglycemic symptoms were occurred. Simultaneously taken blood samples showed the glucose 28 mg/dl, plasma insulin 28.1 μIU/ml, insulin/glucose ratio was 1. The TAUSG and CT scan showed no pathology. In the EUS scanning at the pancreas uncinate process, there was a hypoechoic 11 mm nodular lesion (Figure 2). It was in homogenous echo and well shaped. It was toughed as neuroendocrine tumor. She was examined for multiple endocrine neoplasia. Magnetic resonance imaging (MRI) of hypophysis, hypophysial hormonal analysis and intact parathormone levels were normal. She was scheduled for surgical operation and preoperatively fluid therapy with dextrose in water and 50 μg (tid) octreotide was applied subcutaneously. In the surgical operation the tumor was found at the localization defined by EUS and enucleation procedure was applied. In the histopathologic examination the specimen showed positive staining for insulin and it was in 9 × 9 × 8 mm in size. The glucose and insulin levels were normal at postoperative period.

## Discussion

Diagnosis of insulinoma may delay because symptoms are nonspecific. Sometimes neuroglycopenic symptoms are chiefly seen in patients and these groups of patient are misdiagnosed as epilepsy or neuropsychiatric disease. In patients with insulinoma, diplopia, sweating, palpitation is seen at a rate of 85%; confusion or abnormal behavior 80%; unconsciousness and amnesia 53% and epilepsy 12% [1]. In our cases the major symptoms were sweating, palpitation, tremor and transient unconsciousness. One of them had misdiagnosed as epilepsy previously by neurology clinic. In both cases duration of symptoms was more than one year before diagnosis. Hypoglycemia was mostly seen after exercise and prolonged fasting. In insulinoma cases no other signs or symptoms can be found except hypoglycemic symptoms and obesity. Approximately 25% of cases are overweight because of the hyperalimentation due to hypoglycemic symptoms [5]. One of our cases was over weight and the other had a normal BMI value.

It is diagnosed with hypoglycemic symptoms and hypoglycemia and hyperinsulinism simultaneously. If the blood glucose level is under 40 mg/dl and the insulin level is over 6 μIU/ml, it is diagnosed as hypoglycemia due to hyperinsulinism [5]. If it is suspected of exogenous insulin use, C-peptide levels will be helpful in diagnosis. High levels of C-peptide mark the endogenous insulin secretion. Some different tests can be used in order to show inadequate insulin secretion in cases that are suspected as insulinoma. C-peptide suppression test and prolonged supervised fast tests are two of these tests [3].

80–90% of insulinomas are solid and benign. However they localize on corpus and head mostly, they rarely localize ectopically on pancreas 5–10% of cases are with multiple endocrine neoplasia type I (MEN I) and in this cases the adenomas are generally multiple [1]. The diameter in 90% cases is smaller than 2 cm, in 50% smaller than 1.3 cm, in 30% smaller than 1 cm [6]. One of the research on 30 insulinoma cases has reported that 11 of the their cases were localized on the tail of the pancreas, 6 of their cases were localized on the uncinate process, 6 were on the corpus, 5 were on the head, 2 were on the neck and the dimensions of the adenomas were about 4 – 30 mm (average 13 mm) [7]. Both of our cases had adenoma on uncinate process and the dimensions were smaller than 15 mm and no other clinical or laboratory abnormalities for MEN I suspicion.

A USG, CT, EUS, percutaneous transhepatic portal vein sampling (PTPVS), selective intra-arterial pancreatic stimulation (SIAPS), and also somatostatin receptor scintigraphy (SRS) have been used for tumor localization. But any of the procedures have enough sensitivity. Some of the investigators believe that when insulinoma diagnose clinically and biochemically there is no need to preoperative imaging because most of the lesions were located on pancreas and dimensions were small and also IOUSG and palpation procedures were enough for localization [8]. The localization of the adenoma was important for the decision of surgical strategy and also suggesting to ignore of the nesidioblastosis which is the cause of hypoglycemia by islet cell hyperplasia.

The sensitivity of USG on insulinoma patients were about 29 – 63% because of small dimensions of the tumor and also the fatness of patients as well. In both cases which we underwent, there was no adenoma on pancreas showed in TAUSG, CT is a kind of technique that can determine tumors which are smaller than 1 cm and it is told its' sensitivity is about 40–73% also [2]. Recently it is told that the adenomas that can not be showed with any other techniques could determine some new techniques like dual phase helical CT [9]. The superiority of dual phase helical CT to dynamic sequential scan is the permutation of removing the sequence skipping due to uncooperation of breath-phase and screening of lesion in arterial phase, also. The reason of that is scanning of the pancreas in a breath interval.

Our two cases were underwent CT. Although one of the adenomas diameter was 15 mm – that can be measured by CT – no adenomas were showed in CT anyway. Tumors can be determined with well skilled operators 80 – 90% by EUS [10]. This method is much more successful in tumors which are located in the head of pancreas than located on the tail of the pancreas. EUS imaging chance was found very low on pancreas corpus or tail tumors which are smaller than 1 cm [11]. The advantages of EUS is high frequency resolutions, the very close determination chance to pancreas and elimination of air and bone images. In our two cases the adenomas diagnosed by EUS also.

The localization of adenoma can be determined by measuring the hormones from the branches of portal veins in PTPVS. The diagnostic value is about 60–98%. One of the other techniques is SIAPS. The technique can be explained measuring of hormone from synchronize venous catheter after injection calcium to arterials which are feeding pancreas. Diagnostic value is 90% [12]. Neither TPVS nor SIAPS were used frequently as they are both invasive. As the pancreatic tumors contain so much somatostatin receptors, SRS can be used in diagnose which has 60% sensitivity [13]. SRS is much successful in determining of metastatic tumors than in primer tumors and it indicates that octreotide therapy can be useful in positive cases [14]. In our cases we did not carry out SRS.

IOUSG's sensitivity is about 75–90%. IOUSG and palpation are complementary techniques. Gouyo et al reported that they have diagnosed the tumors 62.5% with inspection and 96.4% with palpation and also they have reported that 20 of 21 patients diagnosed by IOUSG [10]. IOUSG method is especially successful in cases which are not located preoperatively. Because of our cases were diagnosed preoperatively, we did not carry out IOUSG.

Surgery is recommended therapy in insulinomas as the tumors are often soliter and related to benign adenomas. For small and benign characteristic adenomas enucleation is enough and distal pancreatectomy may be suitable for the lesions which are located on the corpus and on the tail of the pancreas. Distal pancreatectomy is suggested for the lesions which can not diagnose by palpation and IOUSG. Total pancreatectomy is suggested for the malign, multiple and large tumors. But medical treatment is suggested when there is contraindication for surgery and in uncontrolled preoperative hypoglycemia. Streptozocin, Diazoxide, Verapamil, Pheytoin, somatostatin can be used for the medical treatment [15].

Both of our cases underwent enucleation. Adenomas have been detected in histopathologic analysis. And both cases were underwent 50 μg (tid) subcutan octreotide treatment.

When EUS is applied by well skilled operators, it can help the chosen operation type as well as there will be no need invasive diagnostic methods. And also intraoperative palpation must be done carefully. However insulinomas localization were pancreas and the tail part of pancreas, they can be diagnosed any other parts of pancreas during the operation. So palpation is much important in these situations. Our findings indicate that a diagnosis can be suggested by biochemical evaluation, clinical symptoms and by EUS.

If there is only one small adenoma enucleation is the most effective method. In this situation complications like diabetes mellitus and exocrine pancreas function disorders can be seen so rarely.

## Consent

Written informed consent was obtained from the patients for publication of this case reports and accompanying images. A copy of the written consent is available for review by the Editor-in-Chief of this journal.

## Abbreviations

EUS: Endoscopic ultrasonography; TAUSG: Transabdominal ultrasonography; CT: Computerized tomography; IOUSG: Intraoperative ultrasonography; BMI: Body mass index; MRI: Magnetic resonance imaging; PTPVS: Percutaneous transhepatic portal vein sampling; SIAPS: Selective intra-arterial pancreatic stimulation; SRS: Somatostatin receptor scintigraphy.

## Competing interests

The authors declare that they have no competing interests.

## Authors' contributions

MO carried out the patient's diagnosis, drafted the manuscript. MFY performed the cases management, drafted the manuscript. EC, BC and MO participated in the patient's management. MFY participated in the writing of the case report. All authors read and approved the final manuscript.
